# Cardiac Failure in Diphtheria

**Published:** 1902-05-10

**Authors:** 


### CARDIAC FAILURE IN DIPHTHERIA.

One of the most important as well as one of the commonest of the toxic effects of diphtheria is cardiac failure. In fact, putting on one side such accidental causes of death as asphyxia or lung disease resulting from involvement of the larynx, one may say that death during the acute toxic stage of the disease invariably arises from primary cardiac failure. A large majority of those who suffer from diphtheria show signs of some degree of heart failure at one time or another during the course of the malady. Dr. Charles Bolton,[1] in discussing this subject, draws a distinction between cases of cardiac failure occurring during (*a*) the acute and (*b*) the convalescent stages.

(*a*) In the acute stage heart failure in the majority of cases sets in a few days before death; and although death may occur on the same day as the signs of failure are first noticed, yet sometimes life may be prolonged for a week or even longer after the symptoms of heart failure have appeared, and all hope of saving the patient has been abandoned. The first sign of failure is noticed in the pulse, which becomes irregular and more compressible. As a rule it is rapid, but sometimes slow, and great slowness is a very fatal sign. The pulse becomes progressively weaker till it is imperceptible, and in this condition the patient may linger for hours or even days, to finally die of syncope. Death, however, may occur quite suddenly if any extra strain is thrown upon the heart, as, for instance, by vomiting or struggling. Besides the irregularity and weakening of the pulse, physical signs of dilatation may be met with in these cases, the cardiac impulse being displaced outwards towards the left anterior axillary line, the impulse being more or less diffuse and slapping in character, and the cardiac percussion note extending a little further to the left than normal, and sometimes a systolic murmur being audible at the apex. The point on which most stress should be laid is the diffuse character of the cardiac impulse and its displacement outwards, especially when it has previously been found in its normal situation.

(*b*) During convalescence heart failure is usually associated with paralysis, and is especially seen in cases of generalised paralysis affecting the palate, pharynx, larynx, and diaphragm. But paralysed patients die from many other causes, thus differing from acute cases in which death is almost exclusively from heart failure. The most terrible cases of sudden death from syncope are those which occur when convalescence is established and the patient is thought to be free from danger. Death under these circumstances is always referable to some strain. Heart failure during convalescence (usually associated with paralysis), is shown by irregularity of pulse with or without signs of dilatation, and it is commonly determined by some strain, such as getting up too early, undue excitement, or vomiting; but the paralysis may be preceded by an irregular pulse, which may in turn have been determined by vomiting.

From a consideration of a number of cases Dr. Bolton says that the very great danger to which patients are liable during the whole course of diphtheria is at once apparent. This danger can only be rendered evident by a thorough and systematic examination of the heart and pulse in every case, however mild, and it can best be guarded against by keeping the patient in bed, or at least perfectly free from all excitement or strain as long as there are any signs of heart failure as shown by an irregular pulse or the physical signs of cardiac dilatation. During the acute stage the chief means of preventing cardiac failure consists in the use of efficient doses of antitoxin at as early a date as possible. Dr. Bolton points out that cardiac thrombosis, to which sudden deaths in diphtheria used to be attributed, is really a rare cause of death in that disease.

[1] Edin. Med. Jour., April 1902.

